# Superb Microvascular Imaging at Evaluation of Nonvisible Buried De-epitelized Flap Vascularization in Breast Reconstruction

**DOI:** 10.5152/eurasianjmed.2023.23063

**Published:** 2023-10-01

**Authors:** Tuğba Gün Koplay, Emine Uysal, Hande Köksal, İbrahim Babalıoğlu

**Affiliations:** 1 Department of Plastic Reconstructive and Aesthetic Surgery, Konya City Hospital, Konya, Turkey; 2 Department of Radiology, Selçuk University Medical Faculty, Konya, Turkey; 3 Department of General Surgery, Selçuk University Faculty of Medicine, Konya, Turkey; 4 Department of Radiation Oncology, Konya City Hospital, Konya, Turkey

**Keywords:** Breast reconstruction, radiotherapy, superb microvascular imaging

## Abstract

**Objective:**

Superb microvascular imaging is a Doppler technique that increases the visibility of small vessels and gives quantitative information about tissue blood supply by measuring the vascular index. In this study, it is aimed to evaluate the long-term and postradiotherapy changes in blood flow of buried de-epitelized flaps in breast reconstruction by using the quantitative values obtained through superb microvascular imaging.

**Materials and Methods:**

Retrospective review of the 14 patients who underwent nipple-sparing breast-conserving surgery and immediately breast reconstruction with a de-epitelized extended latissimus dorsi flap was done. In order to demonstrate the effect of radiotherapy on flaps microvascular circulation, patients were evaluated using superb microvascular imaging postoperative first week, first month, and postradiotherapy first week and sixth month. The normal distribution of the data was evaluated with the Shapiro–Wilk test. Paired samples *t*-test was used for comparisons.

**Results:**

According to the paired samples *t*-tests, postoperative first week mean vascular index was higher than postoperative first month and postradiotherapy first week (*P* < .05). Besides, postradiotherapy first week mean vascular index was higher than postoperative first month and also than postradiotherapy sixth month (*P* < .05).

**Conclusion:**

Radiotherapy can affect the results of breast reconstruction by endothelial and fibrotic injury. In this study, the changes in the microvascular circulation of the latissimus dorsi flap were discussed and found to increase at postoperative and postradiotherapy early period related to inflammation and not decreased significantly at long-term follow-up after radiotherapy.

## Introduction

Breast reconstruction is an indispensable part of breast cancer treatment. While most common technique is implant-based reconstruction, autologous reconstruction has many advantages such as natural results, decreased complications, and increased patient satisfaction, especially for the patients who will receive radiotherapy.^1^ Besides, radiotherapy can lead to fat and flap necrosis. There is no efficient literature about the effect of radiotherapy on microvascular circulation. This study is designed to evaluate the changing of microvascular circulation of the flaps after radiotherapy at early and late periods to contribute to the literature.

The evaluation of flap vascularity is one of the most debated topics in reconstructive surgery. Superb microvascular imaging (SMI) is a Doppler technique that increases the visibility of small vessels through higher resolution and less motion artifact.^2^ Traditional Doppler techniques utilize a 1-dimensional wall filter to remove disarray. This prevents low-velocity blood flow. Superb microvascular imaging uses a multidimensional filter that only removes clutter, thus making slow current signals visible.^3,4^ Superb microvascular imaging has 2 modes as color SMI (cSMI) and monochrome SMI (mSMI). While mSMI suppresses background signals and displays only vessels, cSMI performs simultaneous color coding with grayscale ultrasonography, analogous to traditional Doppler techniques.^5^ Superb microvascular imaging provides quantitative information about tissue blood supply by measuring the vascular index (VI). The percentage ratio of a number of colored pixels in the area of interest to a number of all colored and gray pixels is expressed as VI, a number between 1 and 100.^6^

This study is designed with a standardized group of patients we applied breast reconstruction on, with de-epitelized latissimus dorsi myocutaneous flaps after nipple-sparing partial mastectomy defects. It is aimed to evaluate the long-term and postradiotherapy changes in blood flow of buried de-epitelized flaps by using the quantitative values obtained through SMI.

## Materials and Methods

This study was conducted in conformity with the World Medical Association Declaration of Helsinki. Approval from Karatay University Ethic Committee was obtained (2022/023). The records of the patients with nipple sparing partial mastectomy and reconstruction with buried latissimus dorsi flap were evaluated in terms of microvascular circulation changes at long term fallow-up. Written informed consent was obtained from all participants who participated in this study.

### Patient Selection

Fourteen patients who underwent nipple-sparing, breast-conserving surgery (BCS) by the general surgery clinic and immediately reconstructed with a de-epitelized extended latissimus dorsi flap, that required radiotherapy between 2019 and 2021 were included in this study. Exclusion criteria were previous scar at the donor area, latissimus dorsi flaps with skin island, and patients with missing data. Breast-conserving surgery and sentinel lymph node biopsy were performed by an experienced general surgeon under general anesthesia. In the lateral decubitus position, the extended latissimus dorsi flap was harvested with de-epitelized skin island and transposed through the prepared tunnel to the anterior. All donor areas were repaired primarily. Flap was sutured to glandular margins in patients undergoing breast conservation mastectomy. Radiotherapy was applied at 200 cGy for 25 fractions for the breast and at 200 cGy for 5 fractions as a boost.

Patients were evaluated for the microvascular circulation of flaps by using SMI postoperative first week, first month, postradiotherapy first week, and postradiotherapy sixth month. This method was used routinely because of not being able to visualize skin clinically.

### Image Analysis

**Figure 1. A-D:**
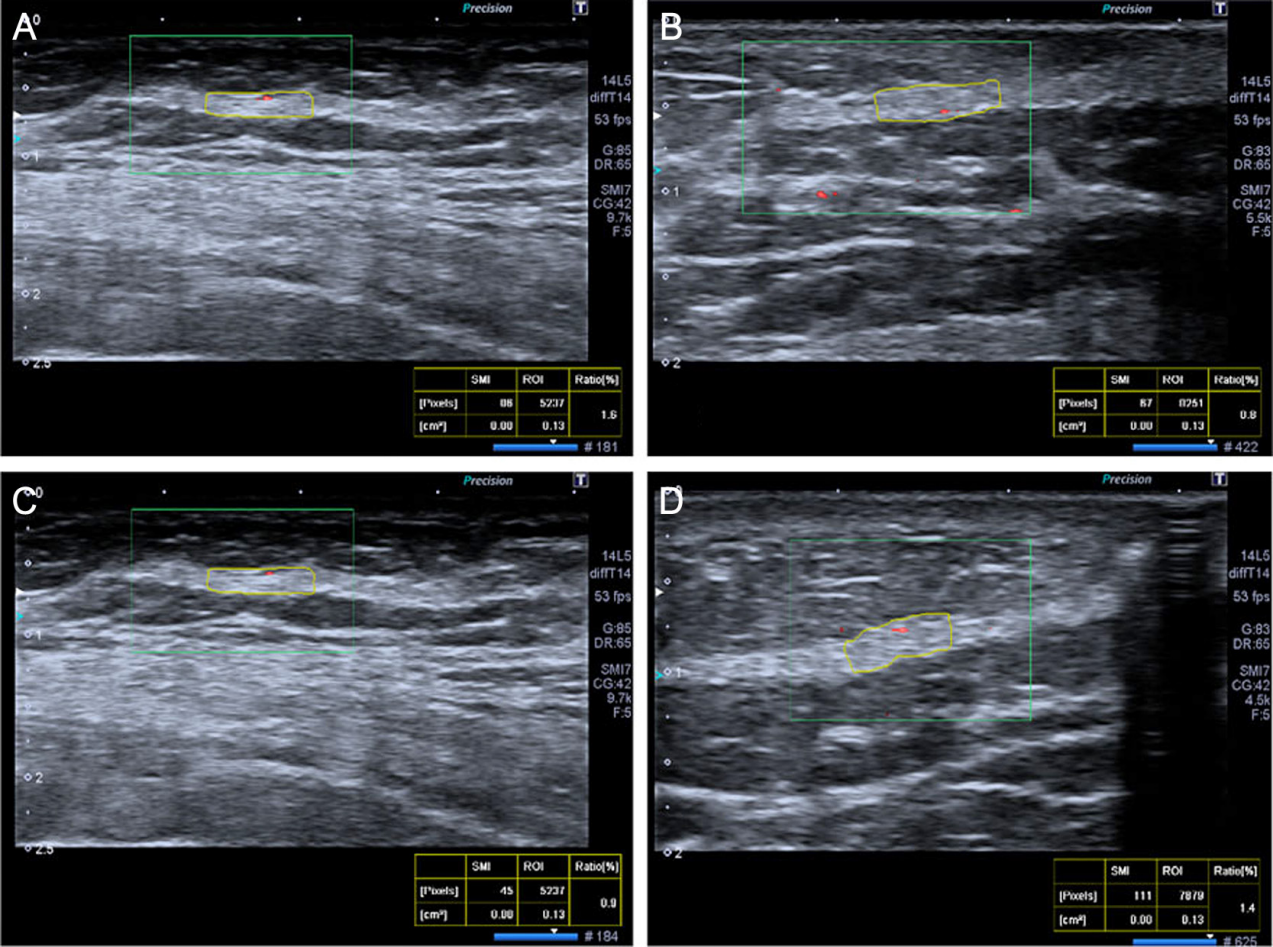
Measurement of vascular index through superb microvascular imaging in standard frame area. (A) Postoperative first week. (B) Postoperative first month. (C) Postradioherapy first week. (D) Postradiotherapy sixth month.

Superb microvascular imaging examination was carried out with the patients in the supine position, with the arm above the head on the same side. During the examination, the patients were asked to breathe superficially and not to move. No pressure was applied to the transducer to avoid collapse in the vessels. For the evaluation of the dermis, the parts with the most intense blood supply were selected. In this plan, VI was automatically measured by the device in an area of 0.13 cm^2^ that was manually demarcated. Thus, dermis blood flow was revealed visually and quantitatively (Figure 1).

### Statistical Analysis

Statistical Package for the Social Sciences-Statistics-version 22 program (IBM SPSS Corp.; Armonk, NY, USA) was used for the statistical evaluation of this study. The normal distribution of the data was evaluated with the Shapiro–Wilk test. Paired samples *t*-test was used for comparisons. The level of error tolerance was maintained at 0.05 (*P* < .05), and decisions were made with a 95% confidence level.

## Results

**Figure 2.**
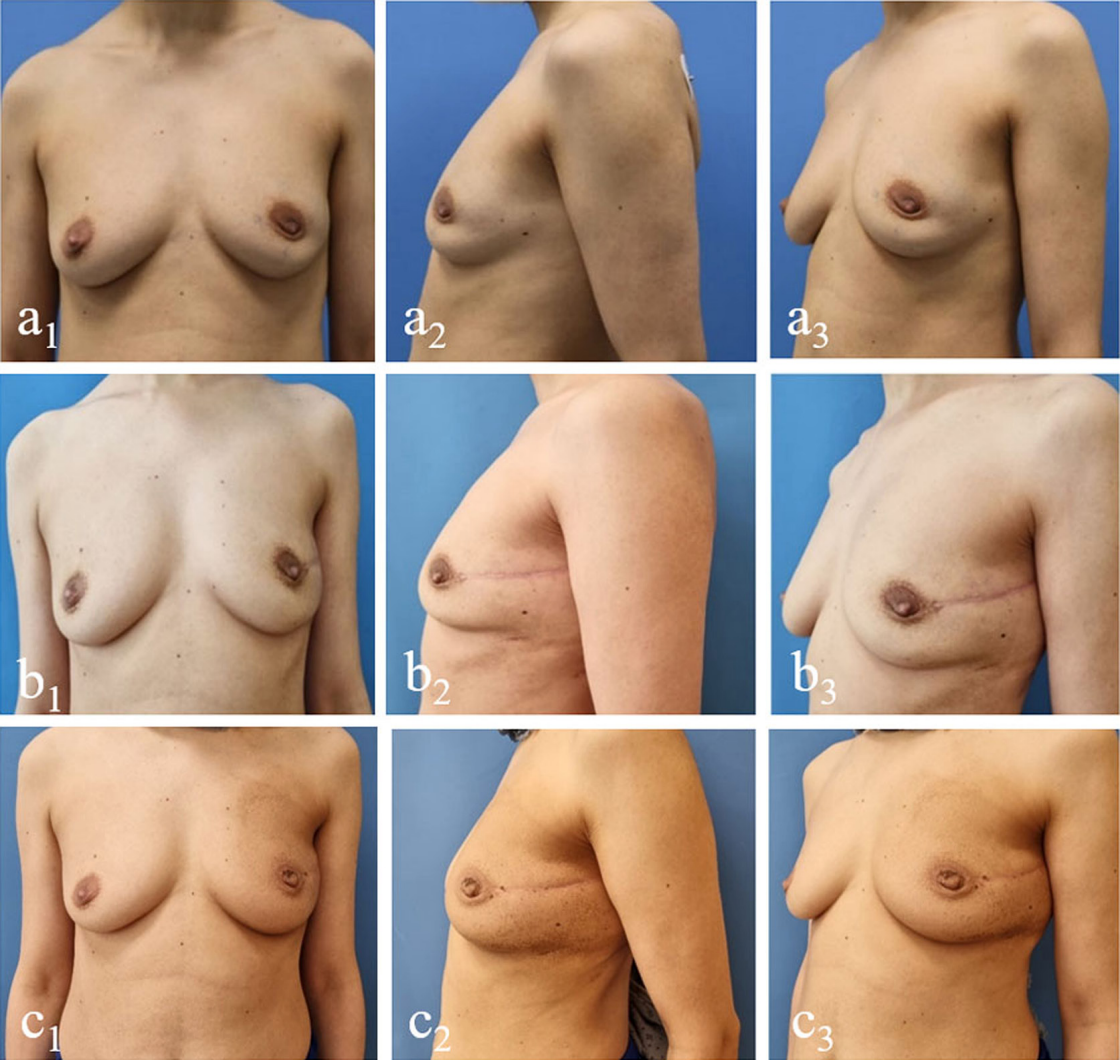
Preoperative (A_1,2,3_), postoperative (_B1,2,3_), and postradiotherapy (_C1,2,3_) images of patients to whom we applied breast-conserving surgery and reconstruction with latissimus dorsi flap.

Fourteen patients with a mean age of 37.7 ± 8.8 (20-56) were included in this study. Nipple-sparing BCS and sentinel lymph node biopsy were applied for all patients and axillary dissection was required for only one patient. All of the breasts were reconstructed with extended latissimus dorsi flap (Figure 2). None of the patients had any comorbidities and none of them were smokers. The average body mass index was 25.4 ± 3.2 kg/m^2^. The mean operation time was 150 ± 35 minutes. The mean hospital stay length was 3 (2-5) days. The follow-up period was 12 ± 4 months. Histopathological results were reported as invasive ductal carcinoma in all patients and ductal carcinoma in situ was detected in 3 patients. The tumor was located at the inner quadrant in 2 patients while at the outer quadrants in 12 patients. Neoadjuvant chemotherapy was applied for 7 patients, and adjuvant chemotherapy and radiotherapy were required for all patients. Patient’s characteristics are shown in Table 1.

**Figure 3.**
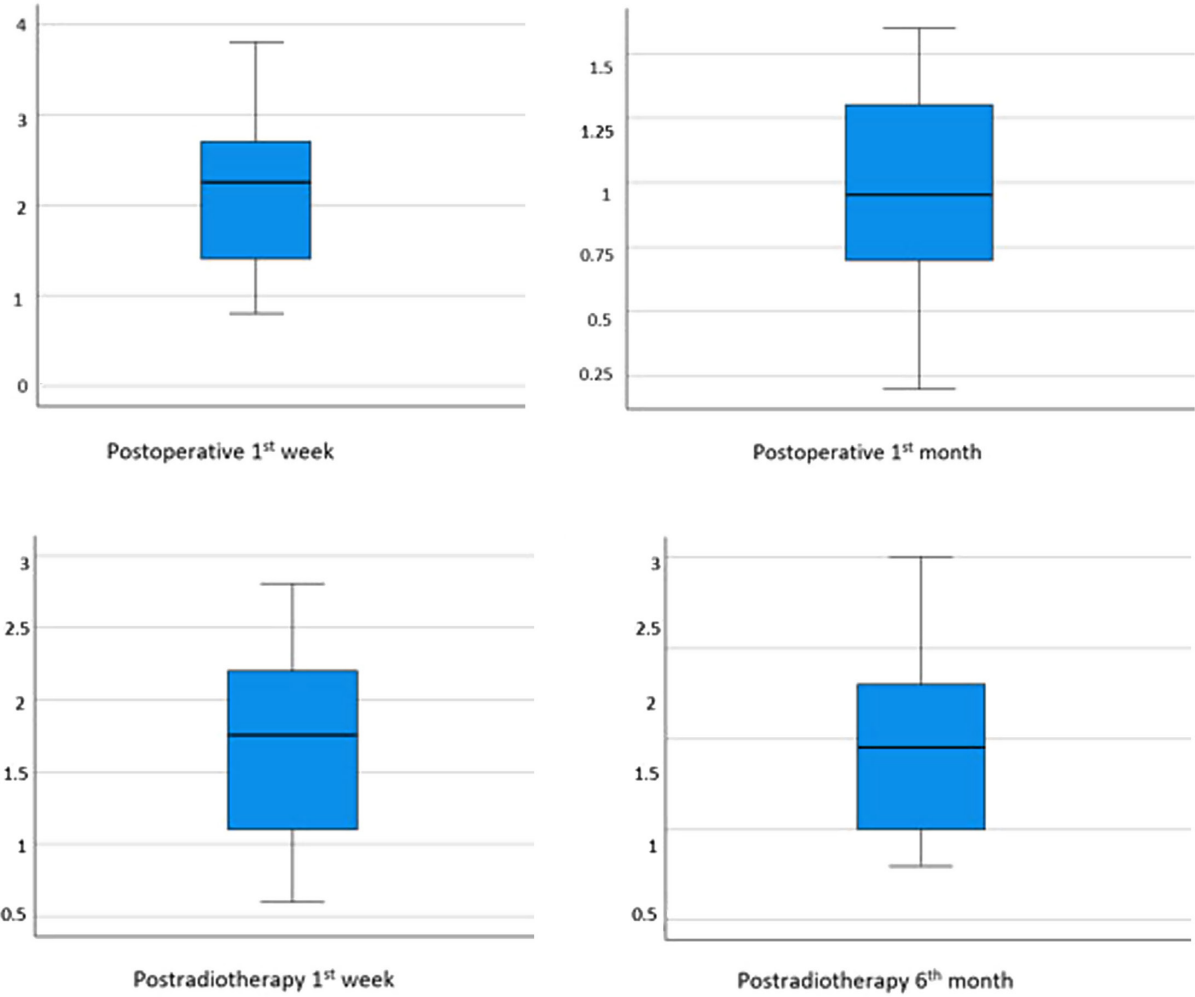
The schematic demonstration of mean vascular indexes at postoperative and postradiotherapy evaluations.

Descriptive statistics were used to compare the general features of all participants. According to the paired samples *t*-tests, postoperative first week mean VI was higher than postoperative first month and postradiotherapy first week (*P* < .05). Besides, postradiotherapy first week mean VI was higher than postoperative first month and also than postradiotherapy sixth month (*P* < .05). On the other hand, there were no significant differences between postradiotherapy sixth month and postoperative first week and first month (*P* < .05) (Figure 3).

## Discussion

Flap monitoring and changes in flap microcirculation relating to different variables have been the subjects of discussion for many studies. It is important to do analyses with objective data in standard populations. In this study, flap vascularization was evaluated with SMI in breast reconstruction without the need for skin island, unlike other techniques. Flap vascularity was found higher in the early postoperative and postradiotherapy periods and decreased in the long term.

Thanks to successful treatments and high survival rates in breast cancer, cosmetic results are now as important as oncological cure in surgical breast cancer treatment. Therefore reconstruction immediately with the BCS as well as skin and nipple-sparing mastectomy are applied frequently with oncologic safety.^7,8^ While the most common technique is reconstruction with implants, autologous reconstruction has many advantages, especially radiotherapy with lower complication rates, higher satisfaction, and better cosmetic results.^9^ For this purpose, latissimus dorsi flap is widely used as a fundamental option for volume replacement in breast reconstruction alone or in combination with implants.

Guidelines for Invasive Breast Cancer from the National Comprehensive Cancer Network recommends adjuvant radiotherapy in cases of 4 or more positive lymph nodes and strongly considered in cases of 1-3 positive lymph nodes, positive margins or margins closer than 1 mm, or tumor > 5 cm in size.^9^ Also, patients with BCS require radiotherapy to reduce recurrence. Consequently, 15% of the patients with breast cancer require radiotherapy.^1^ Radiotherapy leads to volume loss, fat necrosis, wound contracture, fibrosis, and retraction in autologous reconstruction.^7^ On the other hand, Yun et al^9^ found no differences about complication and flap volume between the groups with radiotherapy and without. Fat necrosis related to radiotherapy was shown 2%-52% clinically or radiologically.^10^ Studies suggest that radiotherapy damages proliferating endothelial cells and inhibits angiogenesis,^11^ induces vascular inflammatory changes, resulting in prothrombotic properties of endothelium. Atherosclerosis and venous thrombosis are vascular complications due to radiotherapy. While many studies demonstrated flap and fat necrosis induced by radiotherapy,^9^ there is no literature data about microvascular blood flow changes after radiotherapy. Karimipour et al^11^ showed the potential of lower dose irradiation (100 cGy) to promote neovascularization to improve flap survival by upregulation of VEGF and stimulation of cell proliferation.

In this study, flaps blood flows were detected higher at postoperative first week according to postoperative first month and postradiotherapy first week. Also, it was higher at postradiotherapy first week according to postoperative first week, postoperative first month, and postradiotherapy sixth month. It is thought to be that VI was higher at the postoperative first week and postradiotherapy first week due to inflammation. Statistical differences were not detected between postradiotherapy sixth month and postoperative first month VI values.

In recent years, many novel technologies like Doppler angiography, laser Doppler flowmetry, near-infrared spectroscopy, Indocyanine green angiography, optical coherence tomography, and photoacoustic imaging were published for flap monitoring; however, most of them can not be obtained in every hospital.^12^ The most common and accepted method is clinical observation, but it depends on skin color, temperature, turgor, capillary return, elasticity, etc.^13^ Since de-epitelized flaps do not include skin, monitoring is difficult, skin island can be protected but it leads to a patch image. Ideally, flap monitoring should be cost-effective, simple to be applied, harmless to patient and flap, rapid to obtain, provide reliable and objective data, and be suitable for all types of flaps.^14^ Indocyanin green angiography is a popular option for monitoring but is not standardized.^15^ It provides objective detection of ischemic areas in the operating room.^16^ By detecting the ischemia and preserving complication it lowers the cost but routine usage is not cost-effective.Doppler angiography is another popular technique in vascular imaging but is not sufficient for microvascular evaluation.

Superb microvascular imaging is a radiological technique that can distinguish between low-velocity signals and tissue motion artifacts, showing small vessels in high resolution and detail.^17^ For motion suppression, alterations in the locations of structures are extracted frame by frame, and only the color imaging sections are left. Compared to conventional Doppler techniques, it uses a higher frame rate, and the most prominent advantage of SMI is having images of very fine vascular structures.^18^ The main advantage of SMI in our study is being able to analyze microvascular blood supply of the flap without skin island with quantitative values for detecting the changes due to radiotherapy.

The limitation of this study is being retrospective with a small patient group. Short follow-up is another limitation and new studies can be designed with large groups and long follow-up periods of up to 2 years. The control group could not be planned due to the patient population with BCS and requiring radiotherapy. Also, the effect of chemotherapy could not be evaluated with the control group because all of the patients had chemotherapy.

In the future, SMI can be used for the evaluation of free flaps and in experimental studies about the effects of many variables on the microvascular circulation of flaps. This method gives quantitative data to us about flap vascularization, which is very important for clinical and experimental studies.

In conclusion, as we observed, this is the first report on using SMI as an inexpensive, noninvasive, quantitative alternative for the evaluation of microvascular circulation of the flap. It is observed that flap microvascular circulation is increased at postoperative and postradiotherapy early period related to inflammation and did not decrease significantly at long-term follow-up after radiotherapy.

## Figures and Tables

**Table 1.** Patient’s Characteristics

Parameter	n	%
Neoadjuvant chemotherapy	7	50
Tumor histology		
Invasive ductal carcinoma	14	100
DCIS	3	21
Axillary procedure		
SLNB	13	93
ALND	1	7
Tumor location		
Right superolateral	3	21
Left superolateral	6	42
Right superomedial	2	14
Right inferolateral	3	21
Adjuvant chemotherapy	14	100
Adjuvant radiotherapy	14	100

ALND, axillary lymph node dissection; BCS, breast-conserving surgery; DCIS, ductal carcinoma in situ; NSM, nipple-sparing mastectomy; SLNB, sentinel lymph node biopsy

## References

[1] Halyard MY McCombs KE Wong WW , et al. Acute and chronic results of adjuvant radiotherapy after mastectomy and Transverse Rectus Abdominis Myocutaneous (TRAM) flap reconstruction for breast cancer. Am J Clin Oncol. 2004;27(4):389 394. (10.1097/01.coc.0000071946.11078.7e) 15289733

[2] Uysal E Öztürk M Kilinçer A Koplay M . Comparison of the effectiveness of shear wave elastography and superb microvascular imaging in the evaluation of breast masses. Ultrasound Q. 2021;1;37(2):191 197. (10.1097/RUQ.0000000000000562) 34057918

[3] Park AY Seo BK Cha SH Yeom SK Lee SW Chung HH . An innovative ultrasound technique for evaluation of tumor vascularity in breast cancers: superb micro-vascular imaging. J Breast Cancer. 2016;19(2):210 213. (10.4048/jbc.2016.19.2.210) 27382399 PMC4929264

[4] Hata J . Seeing the unseen. New techniques in vascular imaging. Superb microvascular imaging. Toshiba Rev. 2014:1 8.

[5] Machado P Segal S Lyshchik A Forsberg F . A Novel microvascular flow technique: initial results in thyroids. Ultrasound Q. 2016;32(1):67 74. (10.1097/RUQ.0000000000000156) 25900162

[6] Park AY Seo BK . Up-to-date Doppler techniques for breast tumor vascularity: superb microvascular imaging and contrast-enhanced ultrasound. Ultrasonography. 2018;37(2):98 106. (10.14366/usg.17043) 29025210 PMC5885476

[7] Tran NV Evans GR Kroll SS , et al. Postoperative adjuvant irradiation: effects on tranverse rectus abdominis muscle flap breast reconstruction. Plast Reconstr Surg. 2000;106(2):313 7; discussion 318. (10.1097/00006534-200008000-00011) 10946929

[8] Agrawal A . Oncoplastic breast surgery and radiotherapy-Adverse aesthetic outcomes, proposed classification of aesthetic components, and causality attribution. Breast J. 2019;25(2):207 218. (10.1111/tbj.13193) 30710399

[9] Yun JH Diaz R Orman AG . Breast reconstruction and radiation therapy. Cancer Control. 2018;25(1):1073274818795489. (10.1177/1073274818795489) 30132338 PMC6108018

[10] Garsa AA Ferraro DJ Dewees T , et al. Analysis of fat necrosis after adjuvant high-dose-rate interstitial brachytherapy for early stage breast cancer. Brachytherapy. 2013;12(2):99 106. (10.1016/j.brachy.2012.04.005) 22726878 PMC3929056

[11] Karimipour M Amanzade V Jabbari N Farjah GH . Effects of gamma-low dose irradiation on skin flap survival in rats. Phys Med. 2017;40:104 109. (10.1016/j.ejmp.2017.07.019) 28760508

[12] Deegan AJ Wang RK . Microvascular imaging of the skin. Phys Med Biol. 2019;21(7):07TR01. (10.1088/1361-6560/ab03f1) PMC7787005 30708364

[13] Molitor M Mestak O Pink R Foltan R Sukop A Lucchina S . The use of sentinel skin islands for monitoring buried and semi-buried micro-vascular flaps. Part II: Clinical application. Biomed Pap Med Fac Univ Palacky Olomouc Czech Repub. 2021;165(2):131 138. (10.5507/bp.2021.017) 33821845

[14] Kwasnicki RM Noakes AJ Banhidy N Hettiaratchy S . Quantifying the limitations of clinical and technology-based flap monitoring strategies using a systematic thematic analysis. Plast Reconstr Surg Glob Open. 2021;9(7):e3663. (10.1097/GOX.0000000000003663) 34262835 PMC8274739

[15] Johnson AC Colakoglu S Chong TW Mathes DW . Indocyanine green angiography in breast reconstruction: utility, limitations, and search for standardization. Plast Reconstr Surg Glob Open. 2020;8(3):e2694. (10.1097/GOX.0000000000002694) 32537350 PMC7253278

[16] Kühn F Blohmer JU Karsten MM . Intraoperative indocyanine green fuorescence imaging in breast surgery. Arch Gynecol Obstet. 2020;302(2):463 472. (10.1007/s00404-020-05582-7) 32447448 PMC7321898

[17] Shannon AM Williams KJ . Antiangiogenics and radiotherapy. J Pharm Pharmacol. 2008;60(8):1029 1036. (10.1211/jpp.60.8.0009) 18644195

[18] Carlson GW Page AL Peters K Ashinoff R Schaefer T Losken A . Effects of radiation therapy on pedicled transverse rectus abdominis myocutaneous flap breast reconstruction. Ann Plast Surg. 2008;60(5):568 572. (10.1097/SAP.0b013e31815b6ced) 18434833

